# Disruption of speech motor adaptation with repetitive transcranial magnetic stimulation of the articulatory representation in primary motor cortex

**DOI:** 10.1016/j.cortex.2021.09.008

**Published:** 2021-12

**Authors:** Ding-Lan Tang, Alexander McDaniel, Kate E. Watkins

**Affiliations:** Wellcome Centre for Integrative Neuroimaging, Department of Experimental Psychology, University of Oxford, UK

**Keywords:** Sensorimotor adaptation, Speech motor control, TMS, Auditory feedback perturbation, Speech perception

## Abstract

When auditory feedback perturbation is introduced in a predictable way over a number of utterances, speakers learn to compensate by adjusting their own productions, a process known as sensorimotor adaptation. Despite multiple lines of evidence indicating the role of primary motor cortex (M1) in motor learning and memory, whether M1 causally contributes to sensorimotor adaptation in the speech domain remains unclear. Here, we aimed to assay whether temporary disruption of the articulatory representation in left M1 by repetitive transcranial magnetic stimulation (rTMS) impairs speech adaptation. To induce sensorimotor adaptation, the frequencies of first formants (F1) were shifted up and played back to participants when they produced “head”, “bed”, and “dead” repeatedly (the learning phase). A low-frequency rTMS train (.6 Hz, subthreshold, 12 min) over either the tongue or the hand representation of M1 (between-subjects design) was applied before participants experienced altered auditory feedback in the learning phase. We found that the group who received rTMS over the hand representation showed the expected compensatory response for the upwards shift in F1 by significantly reducing F1 and increasing the second formant (F2) frequencies in their productions. In contrast, these expected compensatory changes in both F1 and F2 did not occur in the group that received rTMS over the tongue representation. Critically, rTMS (subthreshold) over the tongue representation did not affect vowel production, which was unchanged from baseline. These results provide direct evidence that the articulatory representation in left M1 causally contributes to sensorimotor learning in speech. Furthermore, these results also suggest that M1 is critical to the network supporting a more global adaptation that aims to move the altered speech production closer to a learnt pattern of speech production used to produce another vowel.

## 1 Introduction

When we speak, we continuously monitor auditory and somatosensory feedback. If a mismatch between the expected outcome and actual sensory feedback is detected, we use the mismatch to gradually modify our subsequent movements. This form of adaptation is one of the hallmarks of the human speech motor control system, which plays a crucial role in the development and maintenance of speaking skills (Daliri et al., 2018; Maas et al., 2008; Villacorta et al., 2007).

**Fig. 1:**
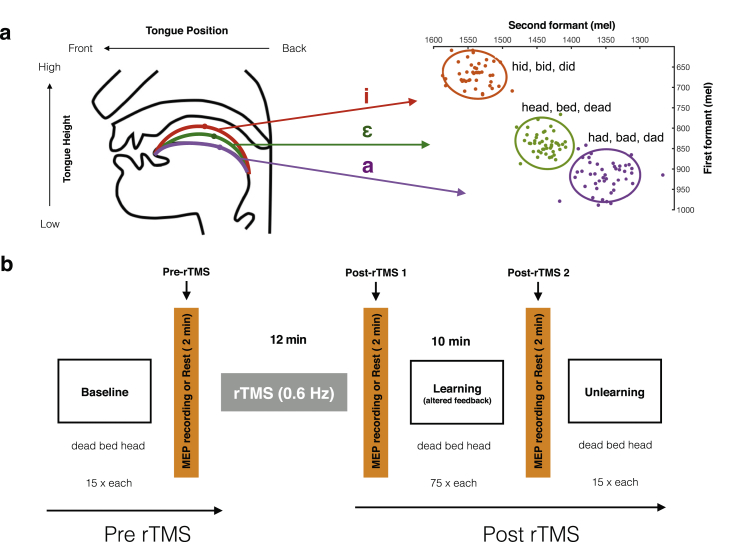
(a) Vowel spaces (adapted from Tang et al., 2019). Schematic showing the relationship between tongue height and position in the vocal tract (left) and the corresponding frequencies of the first and second formants of three vowels (/ε/, /æ/ and /ɪ/) in vowel space from a single representative participant in the current study (right). Data points correspond to F1 and F2 values of single utterances during the vowel exploration. The ellipses represent a 90% confidence interval around the data points of same colour. (b) Experimental procedure. There were three phases of the adaptation experiment: baseline, learning, and unlearning. 12 min rTMS was applied before participants experienced altered auditory feedback in the learning phase. In the hand group only, the changes in cortical excitability induced by the rTMS were measured using single pulses of TMS over the representation of the hand muscle at three time-points: 1) after baseline (pre rTMS), 2) immediately after repetitive TMS (post rTMS 1), and 3) following the learning phase (post rTMS 2) (orange bars). In the tongue group, participants did not receive TMS single pulses and rested for the equivalent time period. In addition, two noise-masked sessions occurred before rTMS and after the final MEP recording/rest block but these data were not included in the analysis (see text for details).

Speech adaptation can be assessed using a real-time perturbation paradigm which has been utilized extensively over the past two decades. A basic type of speech adaptation paradigm involves altering the frequencies of the first (F1) or second (F2) vowel formants or both and playing these back to participants in near real time (Houde & Jordan, 1998; MacDonald et al., 2010). With vowels, the frequencies of the first two formants can largely determine which vowel is perceived. Fig. 1a shows the relative positions of three vowel sounds (/ε/, /æ/ and /ɪ/) in vowel space. In a typical speech adaptation experiment, if the frequency of F1 for the vowel/ε/in “head” is increased, it moves closer to the F1 frequency of the vowel/æ/in “had”. After receiving such altered auditory feedback, participants learn to compensate in their subsequent productions by, for example, lowering F1 frequencies, which would counteract the acoustic change applied, or changing the frequencies of both F1 and F2, which would move their productions closer to a familiar region of the vowel space (vowel /ɪ/ in this case). This compensation occurs over the course of many repetitions without conscious control or awareness (Munhall et al., 2009; Tang et al., 2019).

Production changes resulting from such adaptive learning are retained for a period after the alteration is removed (Lametti et al., 2018a,b; Purcell & Munhall, 2006), suggesting speech adaption involves modification of the feedforward motor commands that guide speech. According to the DIVA (Directions Into Velocities of Articulators) model, the primary motor cortex (M1) receives stored motor commands originating from the feedforward control system (putatively located in the left premotor cortex) and the corrective commands originating from the feedback control system (putatively located in the right premotor cortex and cerebellum), and configures the vocal tract to produce speech sounds (Tourville & Guenther, 2011).

The role of M1 in speech adaptation has received little attention; in contrast, it has been studied extensively in visuomotor adaptation. One recent study explored the possible effects of cerebellar and motor cortex tDCS on speech adaptation induced by real-time feedback perturbation (Lametti et al., 2018a). In response to feedback perturbation of F1, anodal tDCS stimulation over the cerebellum increased adaptation in F1, while anodal stimulation of the ventral motor cortex led to increased adaptation in both F1 and F2. This pattern of findings suggested that the M1 is involved in generating a global adaptation that altered speech production towards the production of another vowel sound rather than compensation simply to offset the acoustic error, which was the pattern of behaviour facilitated by cerebellar stimulation. The electrodes used to deliver tDCS in the previous study were large, however, and covered ventral M1, as well as ventral premotor cortex and may have extended to stimulate portions of posterior inferior frontal cortex.

The current study used repetitive TMS to specifically evaluate the contribution of the articulatory representation to speech adaptation. The perturbation approach provided by rTMS allows greater precision in terms of spatial focality relative to the region stimulated by the large electrodes used in the previous tDCS study (Brasil-Neto et al., 1992; Lametti et al., 2018a). Interference methods, such as the TMS protocol used here, are arguably more convincing tests of causality compared with neuromodulatory methods, such as anodal tDCS, which induced facilitatory effects on learning in the previous study (Lametti et al., 2018a; Scott et al., 2020; Pascual-Leone et al., 1999). The TMS-induced “virtual lesion” approach has several advantages relative to structural lesions in the investigation of causal relationships between brain and behaviour (Walsh & Cowey, 2000). Focal damage to specific brain areas in patients is rare and can lead to functional reorganization over time. TMS-induced disruptions are more focal and temporary, and are not, therefore, confounded by chronic processes mediating functional recovery, thereby allowing investigation of contributions of relatively small brain regions (e.g., motor representations of articulators) to behaviour in the healthy human brain (Hartwigsen, 2015; Möttönen & Watkins, 2012).

We applied a low frequency (.6 Hz) rTMS train to either the tongue or the hand representation in left M1 for 12 min prior to speech adaptation. This train of subthreshold pulses successfully reduces cortical excitability for a further 10–15 min (Möttönen & Watkins, 2009). Previous work has shown that the temporary interference induced by this form of stimulation over speech motor cortex can lead to increased response latencies and decreased accuracy during task performance, and suppressed electroencephalogram (EEG) responses to unattended stimuli (Meister et al., 2007; Möttönen et al., 2013; Sato et al., 2009; Tang et al., 2021). The hand area in left M1 was selected as a control site because: 1) it is close to the tongue area and, therefore, stimulation produces similar levels of noise and scalp sensations to those heard and felt when TMS pulses are applied over the tongue area; 2) it has the same cytoarchitecture (Brodmann area 4; Eichert et al., 2020). However, because of its connectivity, the hand area was not hypothesized to contribute to the speech motor adaptation process that we were targeting. The study adopted a between-subjects design because learning is involved. It should be noted that savings in sensorimotor learning for speech are thought to last at least one month (Houde & Jordan, 2002) and, based on visuomotor adaptation, could last five months and even as long as 12 months (Landi et al., 2011).

We predicted that inhibitory TMS over the tongue representation, but not the hand representation would impair adaptive learning induced by the auditory feedback perturbation. Furthermore, we predicted that temporary disruption of the articulatory representation would impair speech adaptation not only in the shifted formant (F1), but also in the unshifted formant (F2). This prediction was based on findings from previous work indicating that altered auditory feedback of a single formant during speech production induced changes not only in the shifted formant, but also in the unshifted formant (MacDonald et al., 2011), and that neuromodulatory stimulation by tDCS over motor cortex enhances such combined changes (Lametti et al., 2018a).

## 2 Method

### 2.1 Participants

Forty right-handed native English speakers between the ages of 18 and 40 years participated in the main adaptation experiment. Twenty participants received stimulation to the tongue area in left M1 and another twenty participants received the same stimulation to the hand area (control site) in left M1. Demographics of the twenty participants in each of the stimulation groups are given in Table 1.

**Table 1:** Group details (Means ± SD).

	Hand (N = 20)	Tongue (N = 20)	Tongue MEP (N = 5)
Age (years)	22.7 ± 5.2	22.5 ± 5.0	26.8 ± 7.7
Female/Male	10/10	10/10	2/3
Baseline F1 (Mel)	711 ± 86.7	692 ± 83.26	/
Baseline F2 (Mel)	1432 ± 81.0	1448 ± 66.6	/
Active Motor Threshold used for rTMS	48.8 ± 5.6	58.0 ± 5.7	50.0 ± 5.0
Stimulator intensity used to elicit MEPs	53.6 ± 6.1	/	57.4 ± 5.2

Stimulator intensity is given as a percentage of maximum stimulator output.

An additional 5 participants participated in a small proof-of-principle study to confirm the effect of low frequency rTMS over tongue representation on motor excitability as it was not possible to obtain this data at the same time as speech data in the main experiment. All participants reported no hearing problems and no personal or family history of seizures or other neurological disorders. The Central University (of Oxford) Research Ethics Committee approved the experimental protocol. Participants gave informed consent and received payment for their participation. We report how we determined our sample size, all data exclusions, all inclusion/exclusion criteria, whether inclusion/exclusion criteria were established prior to data analysis, all manipulations, and all measures in the study.

### 2.2 Justification of sample size

We previously used the procedure described below to examine the compensatory responses of a separate group of 20 participants when they received a 110-mel increase in F1 feedback (Tang et al., 2019). In that study, participants significantly decreased their F1 production (t_19_ = 6.24, *p* < .001) and increased their F2 production (t_19_ = 2.88, *p* = .005). The effect sizes of these changes were Cohen's d = 1.97 and .91, respectively. Given that the change in F1 is our primary outcome measure of learning, we halved the previous effect size (.98) and determined that even at 90% power, a sample size of 11 would be sufficient to detect a significant (alpha = 5%) reduction in F1 (G∗power 3.1). A group of 20 participants was deemed sufficient in terms of sensitivity to detect a compensatory reduction in F1 production (in response to a 110-mel increase in F1 feedback) of at least medium effect size (Cohen's d = .68), should one occur, 9 times out of 10.

### 2.3 Procedure

During the experiment, participants were seated in front of a computer screen. They were instructed to produce consonant-vowel-consonant words that were presented on the computer screen, into a head-mounted microphone (Shure, WH20) located approximately 4–7 cm away from the right corner of mouth. Each word was presented for 1500 msec, one at a time, with a 750 msec inter-trial-interval. They heard their own voice through over-ear headphones (Sennheiser, HD 280 Pro).

Participants underwent speech adaptation as shown in Fig. 1b. Before the adaptation experiment, participants produced “dead”, “bed”, “head”, “dad”, “bad”, “had”, “did”, “bid”, “hid”, 15 times each with normal feedback to explore the vowel space. Then, during baseline, participants produced three different words “dead”, “bed”, “head”, which contain the same vowel /ε/, 15 times each, again with normal feedback. To induce sensorimotor learning, auditory feedback was altered (see Real-Time Feedback Alteration) while participants produced “dead”, “bed”, “head” 75 times each; we refer to this as the learning phase (10 min). In the final phase, referred to as unlearning, participants produced “dead”, “bed”, “head” 15 times each again with normal feedback as in the baseline.

Participants received the 12-min rTMS train (see below) before the learning phase. Measures of motor excitability were also obtained in the hand group after baseline, before and after learning; the tongue group rested for the equivalent time. Furthermore, two noise-masked sessions were performed after the first and third motor excitability measurements (or equivalent rest), during which participants produced the same words as they produced in the vowel exploration but feedback of their speech was masked by noise that scaled with the amplitude of the produced speech signal (i.e., the signal-to-noise ratio was 0 dB). Unfortunately, the masking was not successful due to a technical problem, so these data were excluded from the experiment.

### 2.4 Real-Time Feedback Alteration

We used a Matlab Mex-based program Audapter (Cai et al., 2008; Tourville et al., 2013) to alter speech and play it back to participants in near real time (e.g., with less than a 40 msec delay). Participants experienced a 110 mel increase in the first formant (F1) production during the learning phase.

### 2.5 Transcranial magnetic stimulation

A low-frequency rTMS train (.6 Hz, subthreshold, 12 min) was delivered over either the tongue or the hand representation of M1 cortex before the learning phase.

All TMS pulses were monophasic, generated by two Magstim 200 sec and delivered through a 70-mm figure-eight coil connected through a BiStim module (Magstim, Dyfed, UK).

After baseline, the hand or tongue representation in the motor cortex was localised using single pulses of TMS. For the hand group, the representation of the first dorsal interosseus muscle in the left hemisphere was localised as previously described (Möttönen & Watkins, 2009). The coil was placed tangential to the skull, to induce a current flow from posterior to anterior under the junction of the two wings of the figure-eight coil. The position of the coil over the lateral scalp was adjusted until a robust motor-evoked potential (MEP) was observed in the contralateral target muscle. After localization, single pulses of TMS were applied over the hand representation to determine the active motor threshold, that is, the minimum intensity at which TMS elicited at least 5 out of 10 MEPs with an amplitude of at least 500 μV when the target muscle was contracted at 20–30% of the maximum. Participants were trained, therefore, to produce a constant level of contraction (20–30% of the maximum) of hand muscles while receiving visual feedback showing the power of electromyography (EMG) activity (more details about EMG signal recording can be found in the following subsection–Electromyography recordings). For the tongue group, we were unable to use tongue MEPs for localisation and to measure changes in excitability before and after the learning phase (as in the hand group), because attaching electrodes to the tongue to obtain an EMG signal would affect a participant's speech production. We, therefore, first localised the hand and lip representations (using EMG electrodes to record from the orbicularis oris muscle; Möttönen et al., 2013) and established the active motor threshold for the lip representation, that is, the minimum intensity at which TMS elicited at least 5 out of 10 MEPs with an amplitude of at least 200 μV when the target muscle was contracted at 20–30% of the maximum. The mean active motor thresholds of each stimulation group are given in Table 1. To estimate the location of the tongue representation, we moved the coil ventrally from the lip representation for half of the distance between hand and lip representations along the trajectory of the line connecting the two. The distance across the scalp between the “hot spots” for tongue and lip representations varied among participants from 12.5 mm to 22.5 mm. The intensity of each participant's active motor threshold was then used for 12 min rTMS (the intensity of active motor threshold for lip representation was used for tongue representation). During the 12-min rTMS train, participants were provided with a silent nature film and were instructed to stay still and relax their muscles (hence the stimulation is subthreshold). During repetitive TMS, the EMG signals were carefully monitored to ensure that muscles were relaxed, and no MEPs were evoked in the target muscle. The coil was changed at the start and halfway through repetitive TMS to prevent overheating.

The success of disruption induced by rTMS in the hand group was evaluated by comparing the size of MEPs elicited by single pulses of TMS over the representation of the target muscle after baseline (pre-rTMS), immediately after the end of the rTMS train (post-rTMS 1), and after learning phase (post-rTMS 2). At each time point, 20 TMS pulses were applied. The intensity used for single pulses TMS was established following localization, as the lowest percentage of stimulator output that elicited MEPs in the target muscle of peak-to-peak amplitude of at least 2 mV on 10 consecutive trials during muscle contraction (see Table 1 for the mean stimulator intensity used to elicit MEPs in the hand muscle). Because we did not record MEPs from tongue muscles in the tongue group (due to interference from electrodes on speech production), single pulse TMS recordings at the three time-points described above were replaced by 2 min breaks (rest). To demonstrate that low frequency rTMS has the same effect on tongue motor excitability as it does on hand, we applied rTMS over tongue representation to another group of 5 participants (tongue MEP group), and recorded MEPs from tongue muscles before (pre-rTMS), immediately (post-rTMS 1) and 10 min after the end of the low-frequency rTMS train (post-rTMS 2). The mean active motor threshold (the minimum intensity at which TMS elicited at least 5 out of 10 MEPs with an amplitude of at least 200 μV when the target muscle was contracted at 20–30% of the maximum) and the mean stimulator intensity used to elicit MEPs (the lowest percentage of stimulator output that elicited MEPs in the target muscle of peak-to-peak amplitude of at least 1 mV on 10 consecutive trials during muscle contraction) for tongue muscles (N = 5) are given in Table 1.

### 2.6 Electromyography recordings

Using a CED 1902 amplifier, a CED 1401 analog-to-digital converter, and a PC running Spike2 (Cambridge Electronic Design), the EMG signal was sampled (5000 Hz), filtered (1 Hz–1 kHz bandpass) and displayed on a computer screen. Participants used this display to practise producing a constant level of contraction of target muscles (20–30% of the maximum). Disposable electrodes were attached to the right orbicularis oris muscle or the first dorsal interosseous muscle of the right hand, to record EMG signal from lip or hand respectively. To record EMG activity from tongue muscles, 2 disposable electrodes were mounted to a noseclip (used by swimmers, Cressi, Italy) and placed above and below the right side of the tongue (Panouillères & Möttönen, 2018). The ground electrode was attached to the forehead for the lip and tongue EMG recordings, and the wrist for the hand recordings.

### 2.7 MEP analysis

MEPs were analyzed trial-by-trial using custom-written MATLAB scripts. For hand MEPs, peak-to-peak amplitude was automatically computed from 15 to 39 msec post-TMS, and for tongue MEPs, peak-to-peak amplitude was automatically computed from 10 to 32 msec post-TMS. The automatic windowing was checked manually by the experimenter. The absolute value of the background muscle activity was averaged across the 100 msec before the TMS pulse and MEPs were excluded from further analysis if a mean absolute value of background muscle activity was greater than the mean plus two standard deviations for each MEP recording session. This resulted in approximately 1.5% of the data from hand group and 2.3% of the data from tongue group (N = 5) being excluded. The mean MEP amplitudes before (pre-rTMS) and after (post-rTMS 1 and post-rTMS 2) disruptive rTMS then compared using one-tailed paired t-tests as the direction of the MEP change was predicted to be a decrease (reduced excitability). The MEP data during post-rTMS 2 for one participant in the hand group was missing.

### 2.8 Analysis of speech data

F1 and F2 frequencies of each utterance were extracted from the data output of Audapter. We confirmed that the Audapter successfully applied F1 frequency increase without changing the fundamental frequency or F2 frequency. Then the average formant frequency was calculated from a window of 30-msec placed over the centre of each utterance. First and second formant values greater than three standard deviations from a participant's mean F1 and F2 values in each phase of the experiment were excluded from further analyses. This resulted in approximately 4.2% of the data being excluded. Four participants (three in the hand group and one in the tongue group) did not complete the unlearning phase due to time constraints.

No significant difference was found between hand and tongue groups in terms of the frequency of F1 or F2 productions during baseline (see Table 1). In each participant, F1 and F2 values were then normalized by subtracting the participant's baseline average (Lametti et al., 2018a; MacDonald et al., 2011; Rochet-Capellan & Ostry, 2011). The normalized results for each utterance, averaged across the participants in each group, can be seen in Fig. 3 (F1 changes) and Fig. 4 (F2 changes).

**Fig. 2:**
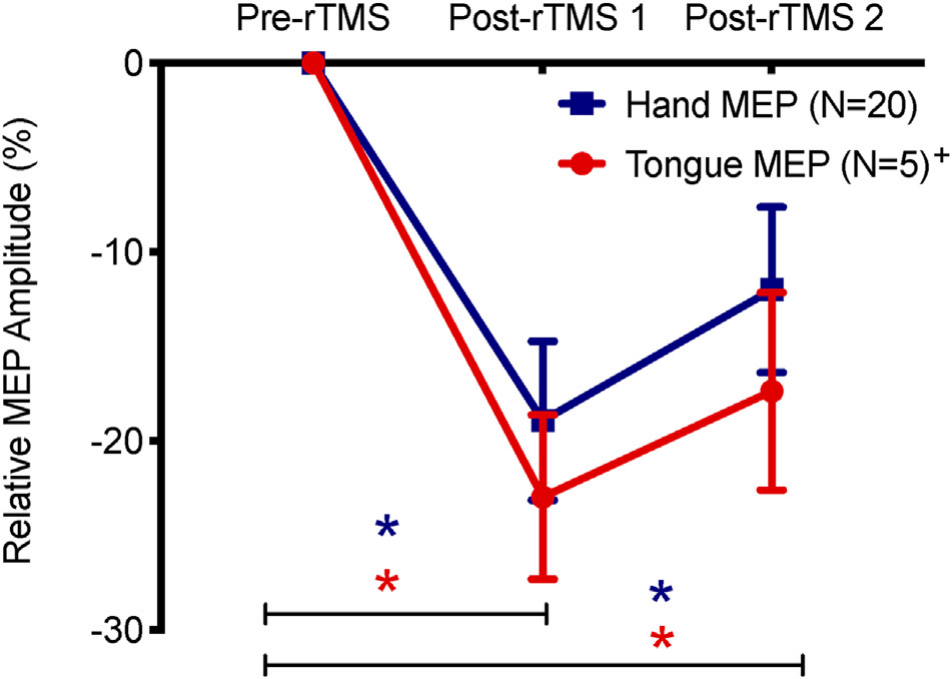
**Effect of rTMS on cortical excitability.** The figure shows mean peak-to-peak amplitudes (±SE) of post-rTMS MEPs (post-rTMS 1 and post-rTMS 2) in relation to pre-rTMS MEPs. MEPs obtained from hand are shown in blue (N = 20) and obtained from tongue are in red (N = 5)^+^. Paired t-tests were used in comparing mean MEP amplitudes obtained in different time points (pre-rTMS *vs* post-rTMS 1; pre-rTMS *vs* post-rTMS 2). ∗*p* < .05. +this was a separate group of participants to the tongue group that completed the adaptation task (see methods).

**Fig. 3:**
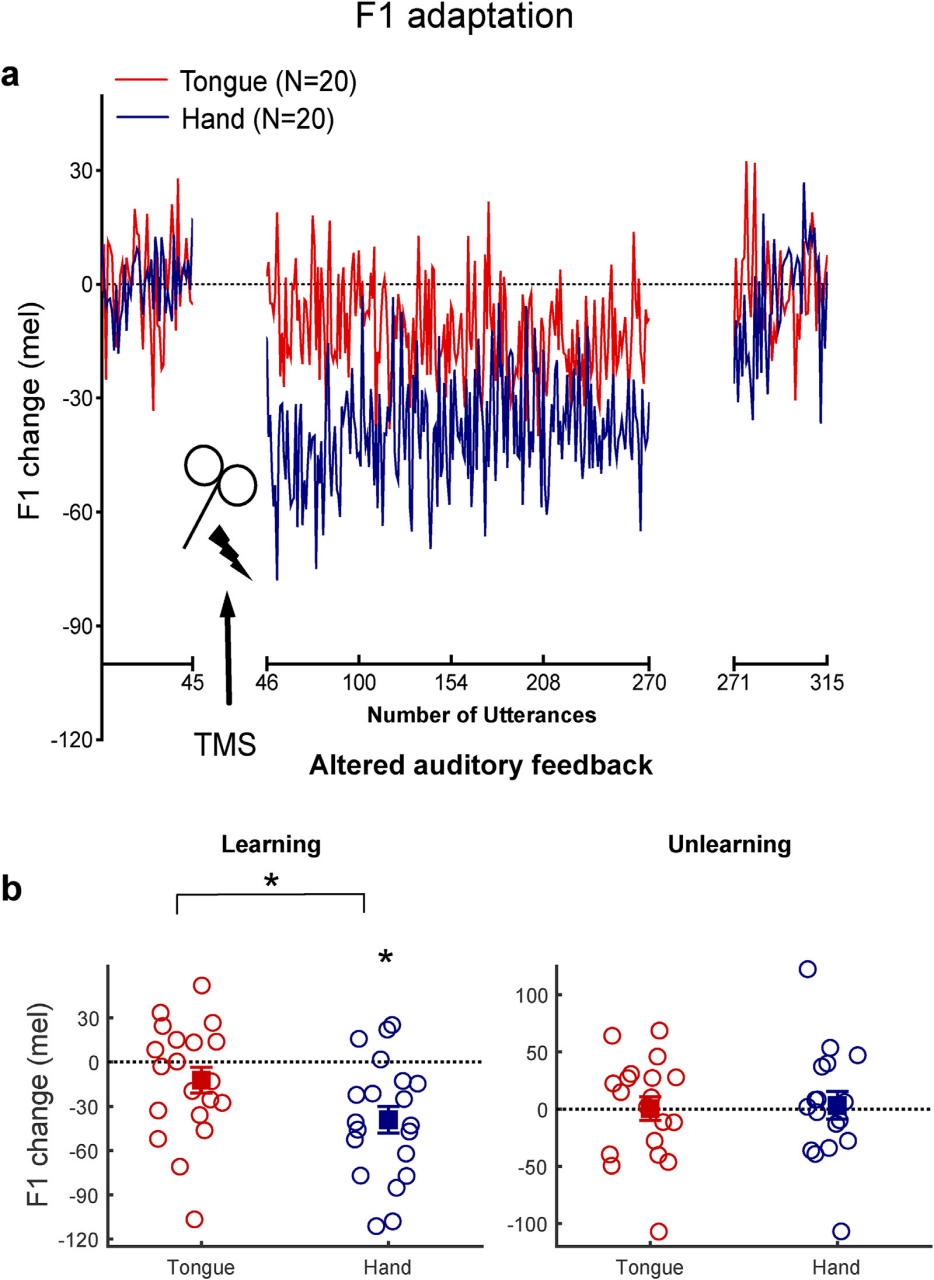
**Changes in F1 production related to sensorimotor learning and rTMS location.** (a) Changes in F1 frequency during baseline, learning, and unlearning phases of the experiment. Solid lines indicate the mean F1 change from baseline for each group. Shaded regions around the solid lines are ± the standard error of the mean. The dotted line at zero is the baseline average (b) Mean F1 changes for the learning phase (utterances 46–270) and the end of the unlearning phase (utterances 301–315). Open circles show the means for individual participants, the filled squares (±SE) represent the group means. Values for hand group are shown in blue and values for tongue group are shown in red. ∗*p* < .05.

**Fig. 4:**
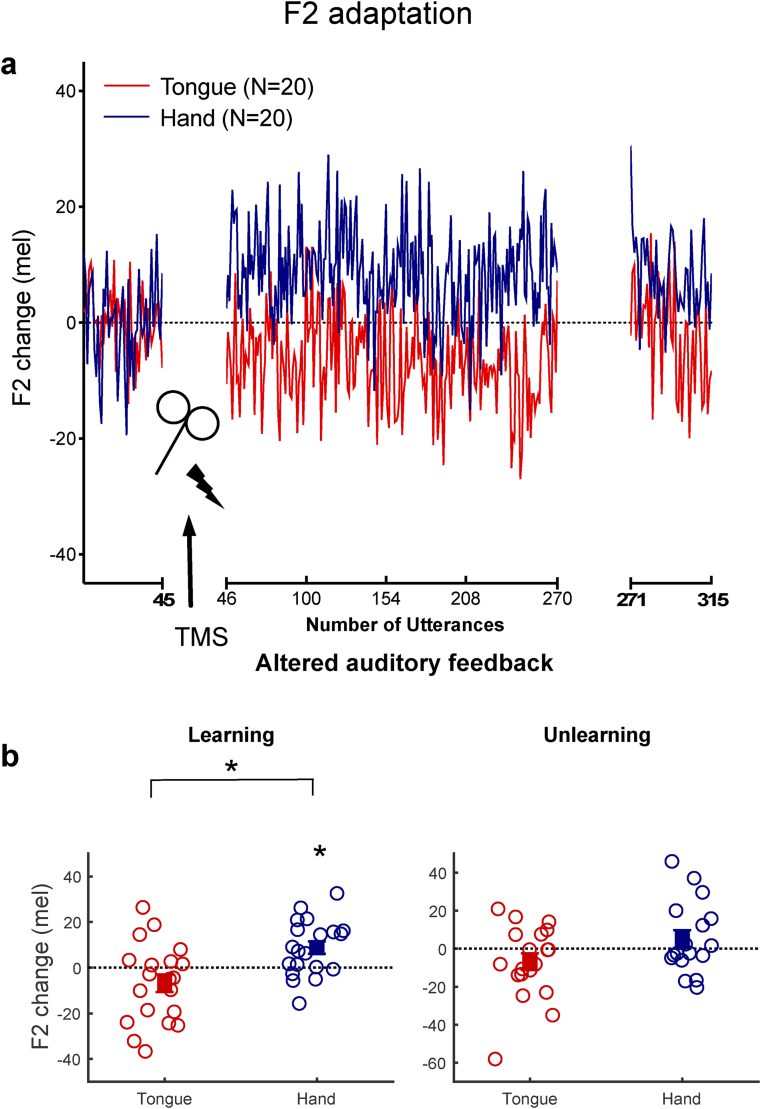
**Changes in F2 production related to sensorimotor learning and rTMS location.** (a) Changes in F2 frequency during baseline, learning, and unlearning phases of the experiment. (b) Mean F2 changes across the whole learning phase (utterances 46–270) and the end of the unlearning phase (utterances 301–315). See legend to Fig. 3 for details.

### 2.9 Statistical analysis

The non-normalized F1 and F2 frequencies during the baseline phase and the learning phase were averaged for each participant and used in the following analyses. Mixed ANOVAs were conducted separately for the F1 and F2 results, with phase (baseline *vs* learning) as a within-subject factor, and group (hand *vs* tongue) as a between-subject factor. Post-hoc comparisons (two-tailed paired t-tests) were only conducted in the event of a significant interaction.

Learning-related changes (baseline-normalized results) during the altered feedback phase were then compared between groups (Tongue *vs* Hand) using a two-tailed t-test. Since null hypothesis significance testing cannot provide evidence for null effects, Bayesian t-tests were performed to test whether the baseline-normalized F1/F2 results during the learning phase were better predicted by the null hypothesis (i.e., the normalized F1/F2 does not differ from zero, the baseline average) or the alternative hypothesis (i.e., the normalized F1/F2 differs from zero, the baseline average) (Lakens et al., 2020).

We also assessed the significance of the return-to-baseline changes in F1 and F2 frequencies in the unlearning phase. The average normalized F1 and F2 changes of the last 15 trials in the unlearning phase were compared against zero (the baseline average) in the hand and tongue groups separately, using a one-sample t-test.

For each participant, we also calculated the Euclidean distance between the centre of the vowel clouds for “hid”, “bid”, “did” produced during the vowel exploration phase of the experiment and for “head”, “bed”, “dead” produced at the baseline and learning phases (Fig. 1a). Learning-related changes in distance were then compared between groups (Tongue *vs* Hand) using an independent t-test.

The effect sizes (Cohen's d) and confidence intervals (CI) around the effect sizes were reported for t-tests. Two-tailed t-tests are reported unless otherwise stated. Bayesian t-tests were performed using the BayesFactor package (version .9.12–4.2, default priors–a two-sided Cauchy √(2)/2) in R (R Core Team, 2019). The significance level for all statistical tests was *p* < .05.

No part of the study procedures or analyses was pre-registered prior to the research being conducted. The raw data generated in this study and analysis codes have been deposited in https://osf.io/26ja8. The data information is summarised in a document available on OSF.

## 3 Results

### 3.1 Effect of rTMS on cortical excitability

Fig. 2 presents the changes in MEP amplitude from baseline (pre-rTMS) at two time-points (post-rTMS 1 and post-rTMS 2) after disruptive rTMS, recorded from hand or tongue muscles in response to single-pulse TMS over the target representations in left M1. Low frequency rTMS over the hand representation successfully suppressed cortical excitability in the hand group. Immediately after the rTMS train (post-rTMS 1), the mean amplitude of hand MEPs was suppressed by 19% on average to 2.13 mV ± SE = .12 (pre-rTMS hand MEPs: 2.71 mV, ± SE = .17; t_19_ = 3.55, *p* = .003, d = .88, CI [.32 1.42]) and after the end of the learning phase (post-rTMS 2), the hand MEP amplitude remained suppressed by 11% at 2.34 mV ± SE = .15 (t_18_ = 2.56, *p* = .021, d = .50, CI [.11 .91]). For the small group of participants (N = 5) in whom we recorded tongue MEPs before and after rTMS, the mean amplitude of tongue MEPs decreased by 23% on average to 1.31 mV ± SE = .24 immediately after rTMS train (pre-rTMS hand MEPs: 1.75 mV, ±SE = .38; t_4_ = 2.66, *p* = .026, d = .60, CI [−.01 1.18]) and remained suppressed by 17% to 1.41 mV ± SE = .26 after an interval of time equivalent to the learning phase experienced by the hand and tongue groups in the main experiment (t_4_ = 2.17, *p* = .048, d = .46, CI [−.07 .96]). Uncorrected *p*-values are reported.

### 3.2 Effect of rTMS on speech adaptation

Fig. 3a shows the group mean F1 change from baseline across the whole adaptation experiment for the hand and tongue groups. During the learning phase, a compensatory decrease in F1 production was clearly observed in the hand group following the introduction of upwards shift in F1 feedback. In contrast, such compensatory changes were disrupted by rTMS applied over the tongue representation, and this pattern of impairment persisted for the remainder of the learning phase. A mixed ANOVA with phase (baseline *vs* learning) as a within-subject factor, and group (hand *vs* tongue) as a between-subject factor was conducted. The analysis revealed a significant interaction between phase and group (Fig. 3a, F_(1, 38)_ = 4.59, *p* = .039, ${\text{η}}_{\text{p}}^{2}$ = .11) as well as a significant main effect of phase (Fig. 3a, F_(1, 38)_ = 17.12, *p* < .001, ${\text{η}}_{\text{p}}^{2}$ = .31). Post-hoc tests revealed that in response to the F1 feedback upwards shift, the decrease in F1 frequencies in the hand group was significant (t_19_ = −4.36, *p* < .001, d = .50, CI [.22 .77]), whereas participants in the tongue group did not significantly change their F1 frequencies from their baseline production (t_19_ = −1.44, *p* = .167).

Learning-related changes (baseline-normalized results) during the altered feedback phase were then compared between groups (Tongue *vs* Hand) using a two-tailed t-test. The F1 decrease in the hand group was significantly larger than that in the tongue group (t_38_ = 2.30, *p* = .028, d = .72, CI [.08 1.36]; Fig. 3b). Bayesian t-tests were performed to weigh the evidence for and against adaptation in each stimulation group by comparing the learning-related changes in produced F1 during the learning phase against zero (the baseline average). For the group receiving stimulation over the hand area, who showed the expected adaptation response, the resulting Bayes factor of 182.68 to 1 in favour of the alternative hypothesis provides very strong evidence for F1 learning in hand group (Jarosz & Wiley, 2014) . In contrast, the group receiving stimulation over the tongue area, who failed to adapt significantly, had a Bayes factor of .56:1 indicating twice as much support for the null hypothesis (i.e., no change from baseline) compared with the alternative hypothesis. These results confirm that subthreshold rTMS over the tongue representation interfered with speech adaptive learning but that this was not due to any effect on movement execution during vowel production more generally.

By the end of the unlearning phase, F1 frequency of production in both groups was at baseline values (Fig. 3b; one-sample t-test against zero, the normalized baseline average; tongue group: t_18_ = .05, *p* = .961; hand group: t_16_ = .28, *p* = .784).

Fig. 4a shows the group mean F2 change from baseline across the whole adaptation experiment for the hand and tongue groups. In response to auditory feedback with an F1 upwards shift, the hand group responded by increasing the frequency of their F2 productions. This response was expected based on previous work using this manipulation (Lametti et al., 2018a; MacDonald et al., 2011; Rochet-Capellan & Ostry, 2011). In contrast, the tongue group exhibited a slight tendency to shift F2 downwards. A mixed ANOVA was conducted and revealed a significant interaction between phase and group (Fig. 4a, F_(1, 38)_ = 11.38, *p* = .002, ${\text{η}}_{\text{p}}^{2}$ = .23). Post-hoc tests revealed that in response to the F2 feedback upwards shift, the increase in F2 frequencies in the hand group was significant (t_19_ = −3.33, *p* = .004, d = .11, CI [.04 .19]), whereas participants in the tongue group did not significantly change their F2 frequencies from their baseline production (t_19_ = 1.74, *p* = .098).

Comparison between groups using an independent t-test confirmed that the F2 changes in the hand and the tongue groups were significantly different (t_38_ = 3.37, *p* = .002, d = 1.07, CI [.40 1.72]; Fig. 4b). Bayesian t-tests were performed to weigh the evidence for and against adaptation in each stimulation group by comparing the learning-related changes in produced F2 during the learning phase against zero (the baseline average). For the hand group, the resulting Bayes factor of 12.22 to 1 in favour of the alternative hypothesis provides strong evidence for F2 learning (Jarosz and Wiley, 2014). In contrast, the group receiving stimulation over the tongue area, who failed to show significant adaptation, had a Bayes factor of .83:1 in favour of the null hypothesis (i.e., no change from baseline). As above, the lack of significant change from baseline values in F2 production confirms that the rTMS over the tongue representation interferes with adaptive responses but does not impair or alter speech production of the target vowel sounds more generally.

By the end of the unlearning phase, F2 frequency of production in both groups was at baseline values (Fig. 4b; one-sample t-test against zero, the normalized baseline average; tongue group: t_16_ = 1.56, *p* = .136; hand group: t_18_ = 1.12, *p* = .278).

As reported in Table 1, the stimulation intensity used in the rTMS train differed significantly between groups: it was higher in the Tongue group (t38 = 5.19, *p* < .0001, d = 1.64, CI [.91 2.35]). To test whether there was a relationship between the intensity used to stimulate the brain and the behavioural effect, we examined the Pearson correlations between rTMS intensity and learning-related changes in F1 and F2 production (baseline-normalised) in both groups. All correlations were below .18 and non-significant (*p* > .05 in all cases). We conclude that there is no relationship between the intensity of stimulation and learning and that a group difference in adaptation cannot be explained by differences in stimulation intensity.

In sum, our findings for the effects of rTMS over the tongue representation in motor cortex on productions of F1 and F2 in response to a F1 feedback perturbation indicate that M1 is critical to the network supporting adaptation.

**Fig. 5:**
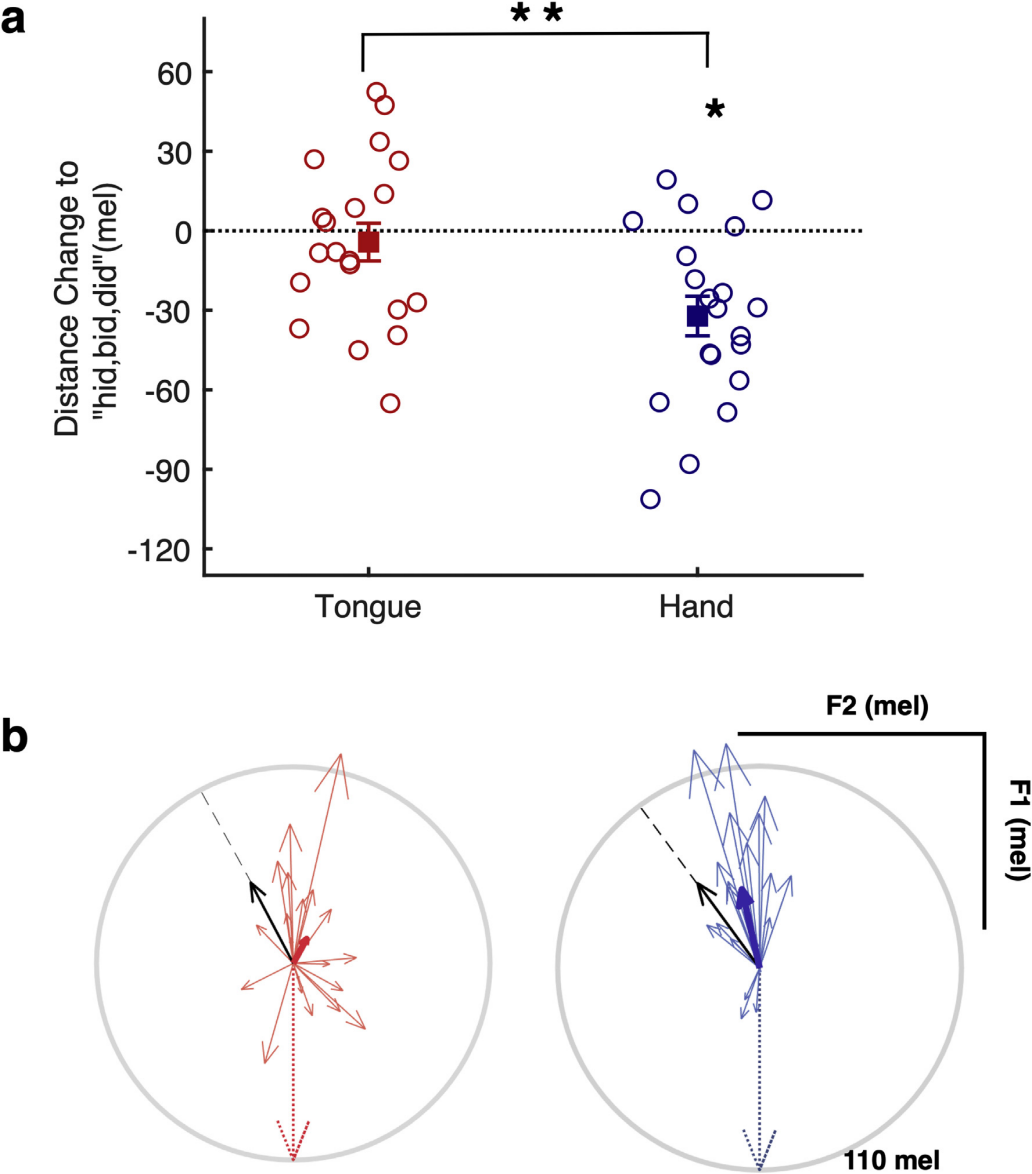
**Coupled F1–F2 changes in vowel space for production during F1 feedback perturbation** (a) Learning-related reduction in Euclidean distance between “head”, “bed”, “dead” and “hid”, “bid”, “did” in relation to sensorimotor learning and rTMS site. Negative values indicate that production of “head”, “bed”, “dead” has moved closer (in vowel space, see Fig. 1) to “hid” “bid” “did” in response to F1 feedback perturbation. Open circles represent data from individual participants, the filled squares (±SE) represent the group means. ∗∗*p* < .01. (b) Vector figures of tongue (red) and hand (blue) stimulation groups. The vectors represent the direction and magnitude of the compensatory change from baseline in vowel space. The thin vectors represent data from individual participants and the thick vectors represent the group average. The black arrow represents the average of the direction required to move from “head” “bed” “dead” to “hid”, “bid”, “did”, while the dotted arrow represents the formant frequency shift we applied. The light grey circle indicates 110 mel (i.e., the magnitude of the shift applied).

Combined changes in F1 and F2, specifically a decrease in F1 coupled with an increase in F2, place utterances closer to values used to produce another vowel sound, namely the /ɪ/ vowel as in “hid”, “bid”, “did” (Fig. 1a). This pattern of compensation was observed previously using a similar paradigm in participants who received sham brain stimulation (Lametti et al., 2018a) and by us using the same paradigm in a separate study without stimulation (Tang et al., 2019). To evaluate how closely the new productions during learning approximated another vowel in the vowel space of individual participants, we calculated the Euclidean distance between the centres of the vowel clouds for “hid”, “bid”, “did” produced during the vowel exploration phase of the experiment with those for “head”, “bed”, “dead” produced at baseline and during the learning phase. In response to the F1 shift-up, the productions of “head”, “bed”, “dead” (across the whole learning phase) in the hand group were 32.17 mel closer to the productions of “hid”, “bid”, “did” in F1–F2 space (one-sample t-test against zero, t_19_ = 4.32, *p* < .001, d = 1.37, CI [.67 2.10]). In our previous study, participants who received no stimulation responded to the same shift-up in F1 feedback by moving their productions of “head”, “bed”, “dead” closer to the vowel space occupied by “hid”, “bid”, “did” by the same magnitude of 32 mel on average (Tang et al., 2019). In contrast to both these groups, for the tongue group, the change in distance (4.27 mel, t_19_ = .60, *p* = .554) was not significantly different from zero (no change). The difference between the hand and the tongue groups in the magnitude of change in distance was significant (t_38_ = 2.71, *p* = .001, d = .86, CI [.20 1.50]; Fig. 5a). Fig. 5b shows the vectors for individual participants indicating the direction and magnitude of compensatory changes in response to the F1 upwards shift (downwards dotted arrow). Participants in the hand group showed the expected combined reduction in F1 and increase in F2 frequencies in response to the altered feedback shifting in the expected direction towards the/ɪ/vowel (blue arrows in Fig. 5b). In contrast the compensatory changes in the tongue group were impaired by rTMS resulting in (mostly) small and inconsistently orientated vectors for individual participants (red arrows in Fig. 5b).

## 4 Discussion

In the current study, we asked whether disruption of the articulatory representation in left M1 by repetitive TMS would interfere with speech adaptive learning. We found that disruption of the tongue representation in M1 impaired the expected compensatory response for the upwards F1 shift in auditory feedback, namely a decrease in F1 frequency alongside an increase in F2 frequency. In contrast, participants who received rTMS over the hand representation showed this expected response. For the participants in the hand group, combined changes in F1 and F2 served to move their productions towards the vowel space representation of the /ɪ/ vowel as previously observed in studies using this paradigm where participants received either sham or no stimulation (Lametti et al., 2018a). The combined F1 and F2 changes made by the tongue group were mostly smaller in magnitude and random in terms of their direction within the vowel space. We suggest, therefore, that the tongue representation in left M1 is one critical part of a network of areas involved in speech adaptation that causally contributes to this process. Below, we discuss our findings in the context of previous work and the putative role of the tongue representation in speech adaptation.

### 4.1 Disruption of articulatory representations in left M1 impaired compensatory responses in the shifted formant (F1)

Our results showed that disruption of articulatory representations in left M1 reduced compensatory responses in the F1 induced by F1 feedback perturbation. F1 compensatory changes in the same speech adaptation task were enhanced by anodal tDCS (which typically increases excitability) of left ventral motor cortex and right cerebellum relative to sham stimulation (Lametti et al., 2018a). A more recent study similarly found that high-definition anodal tDCS of the ventral motor cortex also enhanced compensation in F1 in response to F1 feedback perturbation (Scott et al., 2020). Taken together, the results from these three studies confirm an important role for left speech motor cortex in speech adaptation. The results of the current study extend this previous work by using an interference method rather than neuromodulation, which aims to facilitate behaviour, and is arguably a stronger test of the causal contribution of speech motor cortex to speech adaptation. Furthermore, the TMS used in this study has greater focality than the stimulation used in the previous tDCS study, which used large electrodes covering primary motor, ventral premotor and inferior frontal cortex (Lametti et al., 2018a). In addition, our use of the nearby hand representation as a putatively unrelated control area confirmed that the impairment to the expected compensatory response in F1 was specifically caused by disruption of the tongue representation in left M1.

### 4.2 Disruption of articulatory representations in left M1 impaired compensatory responses in the unshifted formant (F2)

We observed that compensatory changes in F2 were also affected by rTMS applied over the tongue but not the hand representation. This is in line with the previous study in which anodal tDCS over ventral motor cortex enhanced compensatory changes in both F1 and F2 in response to a single formant perturbation (Lametti et al., 2018a). In the current study, these coupled F1–F2 changes produced by participants in the hand group moved new productions closer to values used to produce another vowel sound (i.e., the /ɪ/ vowel as in “hid”, “bid”, “did”; Fig. 1a), a pattern of compensation that was again observed in previous studies using a similar paradigm (Lametti et al., 2018a). We suggest, therefore, that the articulatory representation in left M1 is involved in generating a form of adaptation that aims to move speech production closer to a learnt pattern of speech production used to produce another vowel. It is worth noting, however, that compensatory changes in F2 did not occur in Scott et al. (2020), under either sham or anodal motor stimulation. It is unclear whether this difference reflects small differences between the paradigms, for example the size of the feedback shift or the duration of the learning phase or the large variability among participants. Unlike in studies of visuomotor adaptation, where nearly every participant exhibits compensatory responses after experiencing feedback error (Krakauer et al., 2000), a fairly large proportion of participants in auditory studies shows little or no compensation for auditory perturbations (Lametti et al., 2012, 2014; Rochet-Capellan et al., 2012). These relatively large inter-individual differences suggest that larger sample sizes might be necessary to observe compensatory responses induced by single formant perturbation, especially for changes in the unperturbed formants, which are generally smaller.

### 4.3 In what ways might the articulatory representation in left M1 contribute to speech adaptation?

One possibility is that the left articulatory motor cortex is necessary for motor adjustment to the induced prediction error. In our study, altering the F1 feedback generated a mismatch between the predicted and actual sensory signals, called a sensory prediction error. Over utterances, participants adapted their formant productions to reduce the perceived auditory prediction error, a learning process which is thought to be implicit without conscious control or awareness (Munhall et al., 2009; Shiller et al., 2020). Current models of speech production, such as the DIVA model have attempted to explain the neural network involved in sensorimotor learning in the speech domain (Tourville & Guenther, 2011). According to this model, such prediction error signal is generated in the auditory and somatosensory error maps, which are putatively located in the auditory and somatosensory cortex. In the updated version of the DIVA model, a feedback control map was added, which is assumed to transform the prediction error signals into corrective motor commands, that are later used to modify the forward model (Tourville & Guenther, 2011). Neuroimaging studies suggest that such feedback control maps are located in the right frontal or ventral premotor cortex (Golfinopoulos et al., 2011; Tourville et al., 2008). Our results would further suggest that the left motor cortex is involved in transforming the auditory error signals into corrective motor commands.

The parietal cortex is another brain area considered an integral component for the formation and modification of forward and inverse internal models of motor control based on the limb movement literature (Della-Maggiore, 2004; Wolpert & Kawato, 1998). Accordingly, one previous study used repetitive TMS to demonstrate the contribution of inferior parietal cortex to sensorimotor integration for speech motor learning also (Shum et al., 2011). Participants who received rTMS over parietal cortex exhibited a diminished adaptive response, while the adaptive response of participants who received sham stimulation was unimpaired. It is suggested that the left inferior parietal cortex is also involved in the process of transferring the speech error signal into motor corrective commands, therefore, especially when sensory perturbation is applied.

### 4.4 Comparison with the contribution of M1 to visuomotor adaptation

In contrast to speech motor adaptation, which is largely, if not entirely, driven by implicit changes in motor system, visuomotor adaptation (e.g., reaching) is thought to comprise two dissociable learning components: an implicit process aiming to minimize sensory prediction errors and an explicit process aiming to maintain task goals (Lametti et al., 2020; Mazzoni, 2006). These two processes can work in parallel and eventually drive the acquisition of more accurate motor plans. Compared with the speech motor adaptation literature, the contribution of the motor cortex to visuomotor adaptation has been extensively studied using non-invasive brain stimulation. Findings are inconsistent, however. A relatively large weight of evidence corroborates the notion that M1 is not involved in initial motor adaptation but is important for the retention of learned movement patterns after feedback perturbations are removed (Galea et al., 2011; Hadipour-Niktarash et al., 2007; Richardson et al., 2006). For example, disruption of M1 by repetitive TMS before learning left adaptation to a force field unaffected, but significantly impaired participants’ performances in the same force field 24 h later (Richardson et al., 2006). Similarly, anodal tDCS, which increases neuronal excitability, over M1 had no effect on visuomotor adaptation, but increased the persistence of adapted movements when perturbations were removed (Galea et al., 2011). Conversely, anodal tDCS delivered to M1 during force-field adaptation significantly increased the initial movement trajectory error (in the first 150 msec of movement), which suggests that M1 is involved in the adaptation of reaching movements of human participants (Hunter et al., 2009). A later study revealed that M1 may contribute to reaching adaptation in different phases; disruptive TMS applied to M1 had no effect on the rapid adaptation phase (approximately the first 20 trials when the perturbation was abruptly applied), but reduced adaptation at the plateau later in the learning phase (Orban de Xivry et al., 2011). The findings of the latter study accord with those of studies with non-human primates, which found learning-related changes in the M1 during the late phase of adaptive learning (Paz, 2005; Paz et al., 2003).

### 4.5 Focality of TMS-induced disruption

In general, TMS allows relatively high precision in terms of spatial focality, approximately .5–1 cm (Brasil-Neto et al., 1992; Ravazzani et al., 1996; Sliwinska et al., 2014; Toschi et al., 2008). However, it is challenging to give an exact answer about the volume of tissue stimulated as it depends on the geometry of the coil, skull thickness, stimulus intensity, and the orientation of axons in the cortex under the coil.

It is worth noting that computational simulations of the electrical field induced by TMS are increasingly used to assess the focality of stimulation (Kuhnke et al., 2020; Thielscher et al., 2015). These take individualized models of the head anatomy (e.g., scalp, skull, grey matter etc.) and coil geometry into account (Saturnino et al., 2019) but were beyond the scope of this study since MRI scans of our participants were not obtained. As a rule, the use of lower stimulus intensity results in more focal stimulation (Siebner et al., 2009). In the current study, we applied subthreshold (i.e., low intensity) low-frequency rTMS to the target representation. A concern related to the current study is that TMS over the tongue representation disrupted not only the target region, but also adjacent areas, including premotor and prefrontal cortex. To control for this, and for more general effects associated with TMS, we also stimulated the nearby hand representation in M1 in a separate group of participants; this group maintained the expected compensatory responses to the auditory perturbation. The intensity used to stimulate the hand representation was significantly lower than that used to stimulate the tongue. This was expected due to differences in the distance between the coil and the representations on the cortical surface and has been consistently reported (Möttönen & Watkins, 2009; Rogers et al., 2014; Watkins et al., 2003). We found no relationship between the intensity of the rTMS train and the learning-related changes in F1 or F2 in either group, however. Future studies might consider stimulating a control region with a similar intensity, for example the right hemisphere tongue representation.

It is also worth noting that, although the effects of TMS are focal and maximal at the stimulated area, regions located more distally that are functionally connected to the stimulated site can be affected (Paus et al., 1997). Our results indicate that the primary motor representation of the articulators is a key part of such a functional network. Additional work combining TMS with measures of functional connectivity would shed light on the extent of this network but based on the literature reviewed thus far, it would include inferior, ventral premotor cortex bilaterally, and the cerebellum. Interference at different nodes in this network might produce different types of impairment in speech motor adaptation, which should be the focus of further study.

### 4.6 Low frequency TMS as a tool to reduce cortical excitability of speech motor cortex

The effectiveness of repetitive low-frequency TMS in creating a “virtual lesion” in the area directly under coil was demonstrated in several previous studies (Chen et al., 1997; Möttönen & Watkins, 2009). The MEP size recorded from hand or lip muscles in response to single-pulse TMS over target primary motor representation is significantly reduced after a 15-min train of low-frequency subthreshold rTMS (Möttönen & Watkins, 2009). Our results confirmed the effectiveness of this form of rTMS on temporal disruption of hand motor excitability. We were unable to demonstrate a similar pattern of suppression of motor excitability for the tongue representation concurrent with task performance as placing electrodes on the tongue would have affected speech production. We therefore estimated the position of the tongue representation by localising the hand and lip representations in individual participants. As a proof-of-principle that this estimation was successful and that a 12-min train of subthreshold low frequency rTMS suppresses cortical excitability of tongue representation, we collected MEPs data in a small group of five participants. The results confirmed that the estimation of the location was accurate as MEPs were elicited from the tongue and also the rTMS protocol successfully reduced MEPs size as observed for the hand group.

### 4.7 Limitations

It should be noted that there are a number of limitations to the current study, which affect the inferences that can be drawn based on our findings but which could be addressed in future work. First, we could not rule out the possibility that disruption of the tongue representation in M1 impaired sensorimotor learning by impairing speech perception. Growing evidence (including our own) demonstrates a role for M1 in speech perception (Liebenthal & Möttönen, 2018). Recent TMS studies showed disruption of motor cortex impaired participants' ability to perform a phonetic discrimination task and suppressed their brain responses to changes in speech sounds (Meister et al., 2007; Möttönen et al., 2014; Möttönen et al., 2013; Smalle et al., 2015). In the current study, it is possible that rTMS over the articulatory representation impaired participants’ perceptual acuity, which is positively related to compensatory adaptation (Martin et al., 2018; Nault & Munhall, 2020; Villacorta et al., 2007). Current models of speech production predict that people with better auditory discrimination ability have more distinguishable auditory goals and greater adaptation to auditory perturbations (Houde & Nagarajan, 2011; Perkell, 2012; Tourville & Guenther, 2011). Further work exploring the effect of rTMS-induced disruption over the tongue representation on auditory acuity and how it relates to adaptation could clarify this point.

Second, in the current study, we observed an unusually rapid adaptation in the hand group at the beginning of learning phase (see Fig. 3). Typically, adaptation happens quickly over the first 20 shifted utterances (Lametti et al., 2018a; MacDonald et al., 2011). Then, participants continually adjust their productions at a slower rate. The very rapid learning observed in the current study could be interpreted as a baseline shift induced by brain stimulation. However, further analysis indicated this is unlikely. Firstly, the F1 frequencies of production when the feedback shift was applied were the same as the F1 frequencies for the baseline for the first three trials in participants in the hand group. Secondly, at the end of the unlearning phase when the auditory feedback perturbation was removed, the frequency of F1 productions by the hand group were once again the same as at baseline (see Fig. 3). These results suggest rTMS over the hand area did not induce a baseline shift in speech productions. Nevertheless, our protocol appears to have led to more rapid adaptation at the beginning of learning. One possible explanation of this effect is that TMS has a general arousing or alerting effect leading to faster adaptation in the group receiving stimulation over the control area, and this effect was abolished by stimulation of the target region. Another explanation of the rapid adaptation involves the time interval between baseline and learning phase in this experiment, which did not occur in previous studies (Lametti et al., 2018a; MacDonald et al., 2011; Rochet-Capellan & Ostry, 2011). In one study, a similar length interval between baseline and learning was used to allow completion of a speech perception task (Lametti et al., 2014); participants showed a similarly rapid adaptation to the feedback perturbation. In the current study, the interval between baseline and learning was about 25 min due to TMS localization, thresholding and the 12-min rTMS train during which they were silent. It is possible that the lack of recent normal feedback ‘evidence’ confirming that the sensorimotor system is functioning accurately resulted in a more rapid adaption to the feedback perturbation.

A third limitation of this study is the sample size used for the group comparison. We chose the sample size for each group to be sufficiently powered to detect the learning effect driven by compensation in F1 production due to the F1 feedback perturbation. Because we expected rTMS over the tongue representation would reduce learning, it was critically important to be sufficiently powered to detect this effect. We did not have an a priori power calculation for the difference in learning related F1 changes between groups (Tongue *vs* Hand). Based on the effect size achieved in this study for this comparison (Cohen's d .72), a minimal sample size of 50 (25 in each group) is required to achieve 80% power at an alpha-level .05 (one-tailed) for a future study aiming to replicate this work. It is not unusual, however, for a replication to either reduce the effect size expected or to increase the power and both of these would considerably increase the sample sizes needed.

In summary, we investigated whether the articulatory representation in M1 causally contributes to speech motor adaption. We found that inhibitory TMS over the tongue, but not the hand representation, significantly impaired compensatory changes in both the shifted formant (F1) and the unshifted formant (F2). Importantly, production was unchanged from baseline in the tongue group. Our findings are consistent with the idea that the motor cortex adaptation in the mature system involves changing speech production so that it moves towards previously learned production patterns. We posit that the articulatory representations in left M1 are critical nodes in the network supporting this form of vocal sensorimotor learning and contribute to the process of transforming auditory error signals into corrective motor commands. Such adaptation is important in adulthood for the maintenance of speech production and is presumed to support changes in speech production during development.

## Credit author statement

**Ding-Lan Tang** and **Kate Watkins**: Conceptualization, Methodology, Writing- Original draft preparation, reviewing and editing. **Ding-Lan Tang**: Software, Visualization. **Kate Watkins**: Supervision, Funding acquisition. **Ding-Lan Tang** and **Alexander McDaniel**: Formal analysis, Investigation.

## Open practices

The study in this article earned Open Data and Open Materials badges for transparent practices. The raw data generated in this study and analysis codes have been deposited in https://osf.io/26ja8/?view_only=409275727acc4147a453967890b4f519. The data information is summarised in a word document available on OSF.
